# Self-inflicted penile and testicular amputation: A very rare case report and treatment dilemma in the absence of microsurgical service

**DOI:** 10.1016/j.ijscr.2024.110043

**Published:** 2024-07-20

**Authors:** Mejudin Kedir Abdella, Kaleab Habtemichael Gebreselassie, Henok Ababu Kurabachew

**Affiliations:** a Department of Surgery, Urology unit, Worabe Comprehensive Specialized Hospital, Ethiopia; b Department of Psychiatry, College of Health and Medical Science Dilla University Dilla, Ethiopia

**Keywords:** Genital self-mutilation, Penile/testicular replantation, Case report, Microscopic replantation, Macroscopic replantation

## Abstract

**Introduction and importance:**

Genital self-mutilation is a rare urologic surgical emergency that is usually encountered in patients with underlying psychiatric illness. Because of shortage of published data and variance in management schemes worldwide, these conditions can present a significant management dilemma.

**Case presentation:**

In this case report we present this rare phenomenon, where a known schizophrenic patient presented after he amputated both of his testes and penis under the influence of command hallucination. After 15 h of the incident, macroscopic replantation of the severed genitalia was done and psychiatric evaluation and management initiated simultaneously. But the replantation failed after 9th post operative day.

**Discussion:**

Initial management of patients presenting with genital amputation should be resuscitation. The severed organ has to be washed with sterile saline and placed in “double bag”. There are multiple factors for the success of replantation, the major ones are cooling of the amputated organ and precise microsurgical replantation.

**Conclusion:**

Early intervention and microscopic replantation are crucial for the successful outcome.

## Highlights

• Bilateral testicular and penile self- amputation is one of the rarest forms of self-amputation.
• Prolonged ischemia time has detrimental effect in successful replantation of the severed organ.
• If it is possible replantation of organs should bed done using microscopic surgery.

## 1 Introduction and importance

Scrotal or testicular trauma is uncommon, accounting for only 0.2 % of male trauma patients. Of these penetrating injuries account for the majority compared to blunt trauma. This is explained by the mobility and strong covering of the testes [1,2]. The terms Klingsor Syndrome and Ehsmun Complex are used to describe genital self-mutilation especially in psychiatry patients with religious delusion. It is extremely rare form of self-mutilation. It is usually associated with psychiatric illness and/or with substance abuse. Patients can present with isolated testicular amputation, bilateral testicular amputation or combined testicular and penile amputation. For clinicians these are unfamiliar conditions and they may not have on-site expertise in specialized genitourethral surgeries. And for practicing urologists these conditions can present a significant management dilemma. This is because of shortage of published data and variance in management schemes worldwide [3,4].

Here we present a case report of a male patient who presented after he completely amputated his penis and both testicles under the influence of command hallucination. The work has been reported in line with the SCARE criteria [5].

## 2 Case report

A 28 years old male adult who is a known schizophrenic patient presented to emergency 12 h after he amputated his penis and both testicles. He was diagnosed to have schizophrenia 7 years prior to his current presentation and was on different antipsychotic medications with poor adherence. He is married and has two kids even though his wife abandoned him 5 months before to his presentation. He has a conviction of being sinful and thinks people acknowledge that and talk about it behind his back. Because of this belief he developed depression and hallucination. He also reported he abuses recreational drugs like khat and cigarettes.

**Fig. 1:**
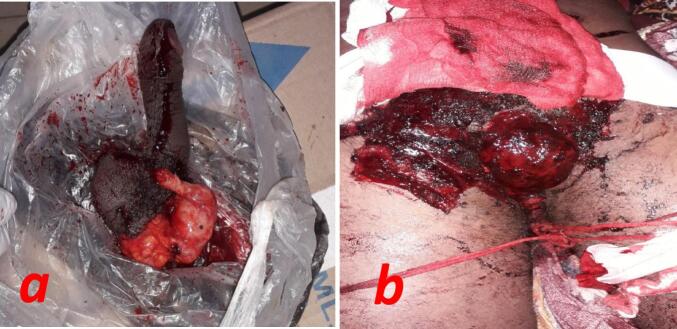
a-Amputated penis and both testes, b-packed perineum with big hematoma covering the amputation base

On the night of the incidence, the patient reported that he saw his three years old son urinating and a voice in his head remind him of his sinfulness and ordered him to cut his genitalia in order to get clean. So, he went to a bathroom and using a blade he amputated his penis and both testicles. He was found in the bathroom after 7 h of amputation. Then he was taken to a local hospital, the wound was packed after the major bleeding vessels were controlled. The amputated genitalia were given to the families in a plastic bag without addition of saline or ice and the patient was referred to our hospital. Up on presentation to our hospital he was confused but conscious. His pulse rate was feeble and 130 BPM, BP = 80/40 mmHg and GCS was 14/15. The perineum was packed with gauze with big clot inside the pack. The amputated penis and testis, covered with the whole scrotal skin, were brought in a plastic bag (Fig. 1a and b).

The patient was resuscitated according to ATLS criteria. CBC was sent and Hg was 12.1 and Hematocrit was 34. After 13 h of genital amputation the urology team was consulted. Up on evaluation the patient was conscious with stable vital signs. Contaminated amputated genitalia was also found in a plastic bag with pink viable looking testis attached to the scrotal wall. The amputated segment was placed in saline bag and that bag was also put in ice slush. Discussion was made with the patient and his relatives on the options of subsequent management. A possible need of repeated operations and the outcome of the operations was also discussed. A written informed consent was taken and after 15 h of the amputation the patient was taken to the operation theater.

**Fig. 2:**
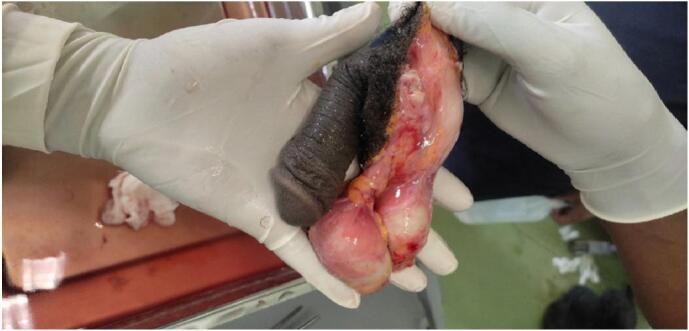
Viable looking testis with broad attachment to scrotal dartos tissue.

**Fig. 3:**
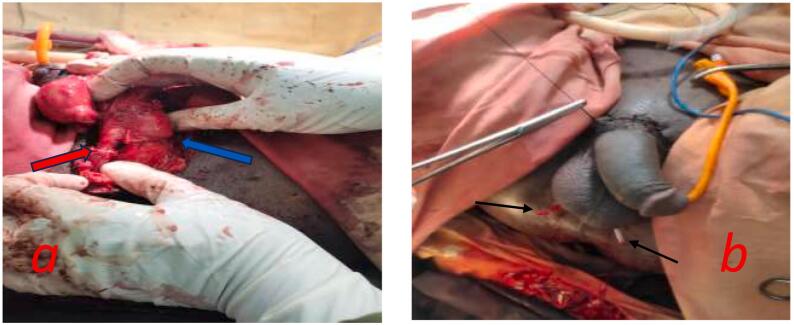
a- After macroscopic replantation of urethra (red arrow) and corpora cavernosa (blue arrow) with 16f foley transurethral catheter in place. b- after approximation of scrotal dartos layer, penile and scrotal skin by placing the testes inside the scrotal cavity. The 2 black arrows indicate the 2 drains in scrotal cavity.

Up on intraoperative evaluation, we found that the penis was transected at the level of mid bulbar urethra. After stiches were removed the corpora cavernosa and spongiosa started to bleed. Both spermatic cords were also identified at the level of external inguinal ring which were ligated with no bleeding. The amputated segments were brought and the dirt was washed with saline. Both testes didn't look ischemic and they were attached by abroad tissue to the scrotal dartos layer (Fig. 2). Macroscopic replantation of the penis done by anastomosing the corpora cavernosa and the urethra after placing 16f transurethral catheter. The scrotal skin and dartos were also approximated by placing the testes inside without microscopic anastomosis of the spermatic cord (Fig. 3a & b). A small tube drain was left inside the scrotal cavity and the procedure was completed with a planned relook after 48 h.

**Fig. 4:**
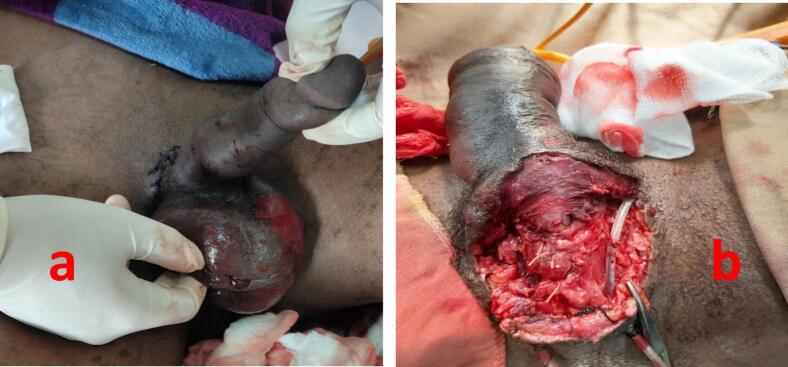
2nd post OP Day: a- shiny edematous scrotal skin with sloughing of epidermis. b- post scrotectomy: intact urethral anastomosis with viable penis.

**Fig. 5:**
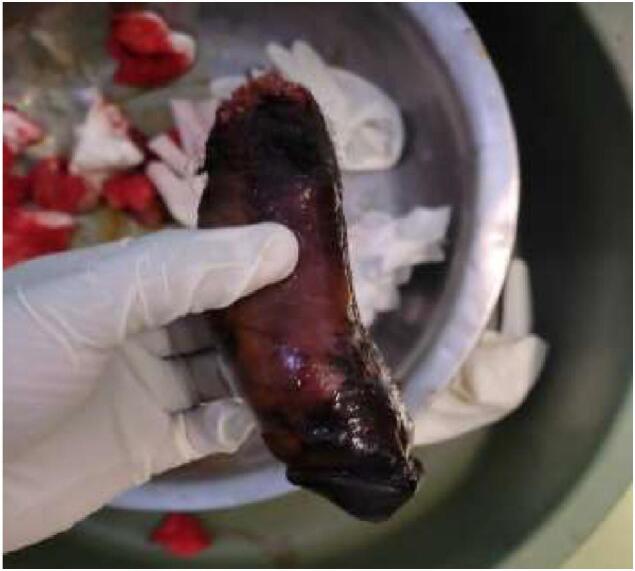
9th Post OP Day: post penectomy rigid, gangrenous penis

Psychiatry consultation was made on the immediate post operative day and olanzapine and diazepam were initiated. Bedside wound exploration was done by releasing some of the stiches after 48 h. The scrotal wall became shinny with ischemic changes. The testes became dark and ischemic but the penis looked viable (Fig. 4a & b). So, the patient was taken to the OR and the testis and scrotal skin were removed, the wound was washed and closed after confirming the urethral anastomosis was intact. On the 9th post operative day the penis became dark, rigid and gangrenous (Fig. 5). The urethral anastomosis also failed and the patient was then taken to the OR and penectomy, debridement and perineal ureterostomy done.

## 3 Discussion

For penile amputation self-mutilation, violence and trauma are the most common causes. On the other hand animal bites, military trauma and self-mutilation are commonly associated with testicular amputation [6,7]. Self-mutilation has been defined as the direct and deliberate self-destruction of part of a person's own body without the intention of suicide. It is often classified into minor and major self-mutilation. The minor being superficial to moderate tissue injury of low lethality such as cutting and stereotypic actions, e.g. head banging. Major self-mutilation is defined as injuries resulting from major traumas and tissue injuries. These are less common but often results in permanent loss of an organ or its function. The 3 main forms of MSM are ocular, genital, and limb mutilation, genital self-mutilation has been among the most dramatic of this class. Major self-mutilation of such severity is so rare that it is hard to predict unless there has been a previous attempt at self-injury, or the patient has spoken about intentions to remove or injure an organ [8,9]. Male genital self-mutilation often encountered within the context of severe psychopathology [10]. Schizophrenia spectrum disorder being the most common underlying psychiatric disorders followed by substance abuse disorders, personality disorders, and gender dysphoria [8]. The subject in our case study is diagnosed to have schizophrenia with command hallucination. We believe that his recent separation from his wife and poor adherence to his antipsychotic medications seems to precipitate this drastic measure.

Initial management of patients presenting with genital amputation should be resuscitation. Then, if the severed genitalia are recovered and not too badly damaged, the patient should be prepared for immediate re-plantation. The severed organ has to be washed with sterile saline and placed in “double bag”, where the severed organ should be wrapped in saline soaked gauze and placed in sterile bag and that bag also should be placed in another bag which contains iced water. It is also important to avoid direct contact between the severed organ and the iced water to prevent hypothermic injury. If penile stump is long enough to be clamped it should be clamped to avoid excessive blood loss. In order to avoid damaging the vessels and causing loss of length, it is wise to use ligatures or long cut suture ligations. If that is not feasible a pressure dressing should be applied until replantation or proper hemostasis is secured [11,12]. In our case the patient was referred to our hospital after hemostasis was secured but he was still in shock and the severed genitalia was not washed nor placed in “double bag” technique. In our hospital proper resuscitation was done, the severed genitalia was washed placed in “double bag” technique and compressive dressing was applied until the patient was taken to the operation theater.

There are three ways to manage penile amputation depending on the amputated penis. The first option is closure of penile stump and doing urethrostomy after spatulation. This is usually done if the amputated penis is not available. The second option is surgical replantation of the amputated penis. The third is total phallic replacement. Successful replantation of amputated penis is possible after 16 h of cold ischemia time or 6 h of warm ischemia time. But trial of replantation should be performed with in 24 h of amputation. For the testes because of the high metabolic state, it has only 4 to 6 h for successful replantation [12,13]. Ischemia time of 6 h result in permanent loss of spermatogenesis. Interestingly endocrine function has been observed after 16 h of ischemia [14].

There are two ways for replanting an amputated penis: microscopic and macroscopic. The best surgical outcome is achieved if replantation is done in a well experienced multispecialty center using a microscopic technique. If such a facility is unavailable, macroscopic anastomosis of the urethra and corporeal bodies should be performed [4,12]. Replantation of a severed testes is a very challenging procedure and it should be done microscopically. It involves reconstruction of testicular artery, pampiniform plexus, and vas deferens microsurgical anastomosis. The artery of the amputated testicle can be small and difficult to differentiate it from the vein [4].

Our patient was taken to the operation theater after 15 h ischemia time. That is 11 h of warm ischemia time and 4 h of cold ischemia time. Which was way too long for the testis to regain its full function if replanted. The time is also a bit long for replantation of the penis but still it can be tried. Because of lack of microsurgical service testicular replantation was not done. But at the time of operation, the testis looked viable with good and broad attachment to the scrotal skin with dartos tissue. Even if testicular ischemia was in evitable because of the above finding, we left the testis in scrotal cavity for second look and we approximated the dartos tissue and the scrotal skin after we left a drain. The penis also replanted in macroscopic technique because of lack of microsurgical service.

There are multiple factors for the success of replantation, the major ones are cooling of the amputated organ and precise microsurgical replantation [14]. The failure of penile replantation in our patient can be explained by long ischemia time and the use of non-microsurgical technique.

## 4 Conclusion

This study highlights the rarity of genital self-mutilation especially that involves testicular amputation. The limited availability of publication and unfamiliarity of physicians to these case makes the management complex. The outcome of the replantation of the severed genitalia is highly dependent on early intervention, microscopic replantation and multidisciplinary approach.

Written informed consent was obtained from the patient for publication of this case report and accompanying images. A copy of the written consent is available for review by the Editor-in-Chief of this journal on request.

## Ethical approval

Case reports are not constituted as research in our hospital, so it is exempted from ethical approval in our institution by Worabe Comprehensive Specialized Hospital IRB.

## Funding

We did not get any funding for preparation of this case report.

## Author contribution

Mejudin Kedir

• Study concept and design
• Data collection
• Data curation
• Literature review
• Writing paper

Kaleab Habtemichael

• Journal selection
• Study concept and design
• Review and editing

Henok Ababu

• Data collection
• Data curation
• Literature review

## Guarantor

Mejudin Kedir.

## Research registration number

1. Name of the registry: Clinicaltrials.gov.
2. Unique identifying number or registration ID: NCT06448741.
3. Hyperlink to your specific registration (must be publicly accessible and will be checked): https://clinicaltrials.gov/ct2/show/NCT06448741.

## Conflict of interest statement

All authors have no conflict of interest.
